# Prevalence of Acute Myocardial Infarction and Changing Meteorological Conditions in Iran: Fuzzy Clustering Approach

**Published:** 2020-05

**Authors:** Hamid SHARIF NIA, Ozkan GORGULU, Saeed PAHLEVAN SHARIF, Erika Sivarajan FROELICHER, Ali Akbar HAGHDOOST, Samad GOLSHANI, Ameneh YAGHOOBZADEH, John Henry NOBLE, Roghieh NAZARI, Amir Hossein GOUDARZIAN, Farhad AREFINIA

**Affiliations:** 1. Department of Nursing, Amol School of Nursing and Midwifery, Mazandaran University of Medical Sciences, Sari, Iran; 2. Department of Biostatistics and Medical Informatics, Faculty of Medicine, Ahi Evran University, Kırşehir, Turkey; 3. Taylor’s Business School, Taylor’s University Malaysia, Subang Jaya, Malaysia; 4. Department of Physiological Nursing, School of Nursing, University of California San Francisco, San Francisco, California, USA; 5. Department of Epidemiology & Biostatistics, School of Medicine, University of California San Francisco, San Francisco, California, USA; 6. Research Center for Modeling in Health, Institute for Futures Studies in Health, Kerman University of Medical Sciences, Kerman, Iran; 7. Department of Cardiology, Cardiovascular Research Center, Mazandaran University of Medical Sciences, Sari, Iran; 8. Department of Nursing, School of Nursing and Midwifery, Tehran University of Medical Sciences, Tehran, Iran; 9. Board of Directors Alliance for Human Research Protection (AHRP), New York, NY, USA; 10. Student Research Committee, Mazandaran University of Medical Sciences, Sari, Iran; 11. Amol School of Nursing and Midwifery, Mazandaran University of Medical Sciences, Sari, Iran

**Keywords:** Meteorological parameters, Seasonal changes, Acute myocardial infarction, Prevalence

## Abstract

**Background:**

The prevalence of Acute Myocardial Infarction (AMI) varies from region to region caused by seasonal climate changes and temperature variation. This study aimed to assess the relationship between changing meteorological conditions and incidence of AMI in Iran.

**Methods:**

This retrospective prevalence study was based on medical records of the heart center of Mazandaran Province on all patients diagnosed with AMI in Mazandaran, northern Iran between 2013 and 2015. Patients’ sex and the day, month, year and time of hospital admission were extracted from patients’ records. Moreover, the meteorological reports were gathered.

**Results:**

A statistically significant difference was found between the distributions of AMI cases across 12 months of the year (*P* < 0.01). Fuzzy clustering analysis using 16 different climatic variables showed that March, April, and May were in the same cluster together. The other 9 months were in different clusters.

**Conclusion:**

Significant increase in AMI was seen in March, April and May (cold to hot weather).

## Introduction

Cardiovascular diseases (CVDs) are the most common noncommunicable chronic disease (1) and the leading cause of death in the world, Middle East region, and Iran (2). Death from CVDs reaches 25 million in 2020 and will rise to more than 23.6 million in 2030 (3). One of the most important types of CVDs is acute myocardial infarction (AMI) that leads the most range of death (4). AMI is a common cause of hospitalization and mortality that affects 1.2 million people in the world annually (4, 5). AMI has increased 12 times in women and almost 14 times in men from 1990 to 2020 in developing countries like Iran (6). According to the Census of Health Ministry and Medical Education of Iran, 39.3 percent of deaths are related to heart disease, and 19.5 percent of them is attributable to AMI (2).

Although risk factors such as smoking, obesity, high blood pressure and diabetes are known causative agents of AMI. Epidemiological studies also have highlighted an increased incidence resulting from climate changes (7). AMI incidence varies from region to region caused by seasonal climate changes (summer and winter) and temperature variation (8). This widespread phenomenon was first recognized by Hippocrates in his treatise “Of Airs, Waters and Places” (9). Thus, the incidence of AMI and temperature changes associated with the changing seasons occur uniformly throughout the world (10).

Most studies have reported that the highest incidence of AMI occurs during the winter season (11–13); and the reduction in temperature by 1 °C is significantly associated with an increased incidence of AMI (14). However, the problem is more prevalent in summer (15–17). Yet another study identified spring as the peak time for increases in hospitalizations of patients with AMI (18). Some studies have examined separately the influence of meteorological factors on AMI (19, 20), in addition to temperature and climatic conditions such as humidity, wind speed, and air pressure affecting the incidence of AMI (21, 22). Therefore, there is uncertainty about the relationship between specific meteorological conditions and the risk that these conditions pose on AMI (23, 24).

Changes in meteorological conditions in different countries lead to different levels of hospitalization and mortality due to AMI (25). Some studies implicate winter and summer as the highest and the lowest seasons for hospitalization and mortality caused by AMI, respectively (25, 26). Report the admission rate of AMI to the heart centers of Isfahan, Iran increased from the low in autumn (4.2%) to high in winter (15.8%) and spring (17.8%).

Since there is only one study (25) examined meteorological conditions and the risk of AMI in Iran, this study seeks to enhance the knowledge of this phenomenon in the northern region of Iran in 2016.

## Materials and Methods

This study was based on medical records of the Heart Center of Mazandaran Province, northern Iran (36.369 N, 52.270 W) from 2013.01.01 to 2015.12.29. This center provides the most comprehensive data in northern Iran on all patients with a diagnosis of AMI.

### Setting

Sari City (the provincial capital of Mazandaran) is in the north of Iran with a mild climate. It has a population of 505,000 inhabitants according to the latest census in 2016 (27). The study site was the Professional Heart Center of Fatemeh-Zahra, Sari, Iran. It has six Coronary Care Units (CCU), one ICU, and one emergency wards. Recorded data of the patients with AMI were obtained from these units.

### Study population

All the patients with symptoms of AMI admitted to hospitals during 2013–2015 has been included in the current study. The research team consisting of one cardiologist, two registered nurses with experience of working in cardiac unit for at least 12 years, a statistician and an epidemiologist to extract and record the needed information. The final diagnosis of AMI was recorded by the cardiologist. Confirmatory diagnostic criteria were: 1) presence of cardiac enzymes (CK or CPK-MB) above the normal range; 2) elevation or depression of ≥1 ST-segment; 3) abnormal Q waves; and 4) presence of Troponin enzyme in the blood.

After obtaining approval of the Mazandaran University of Medical Sciences, the two nurses approached patients with a confirmed AMI diagnosis to the selected Heart Center to extract and record patient data using existing electronic medical records.

### Measurements abstracted

Patients’ sex and the day, month, year and time of hospital admission were extracted from patients’ records. The Meteorological Organization of Iran provided information from Jan 2013 to Dec 2015 about daily temperatures (Celsius) (minimum, maximum, and average), average temperatures, average wind direction, wind direction 6.5 AM, wind direction 12.5 PM, wind direction 18.5 PM, average wind speed, wind speed 6.5 AM, wind speed 12.5 PM, wind speed 18.5 PM, and its direction, rainfall (day), daily evaporation rate (mm), sunshine duration (hours a day), average relative humidity, relative humidity 6.5 AM, relative humidity 12.5 PM, relative humidity 18.5 PM, and relative humidity (percent). Iran’s four climate seasons are spring (April to June), summer (Jul to Sep), autumn (Sep to Dec) and winter (Jan to Mar) (28).

### Ethics approval

The study was approved by the Ethics Committee of associated University of Medical Sciences (Ethical Code: IR.MAZUMS.REC.96-10232), Sari, Iran, according to its code of ethics, including assured confidentiality of all patient information.

### Statistical analysis

The meteorological variables included were the above mentioned. S-Plus 2000, SPSS ver. 20 (Chicago, IL, USA) and MATLAB 14 statistical programs were used to analyze data. A fuzzy clustering analysis by following the method suggested by Bezdek, Hathaway (29) and Kaufman and Rousseeuw (30) was conducted to determine if there was an association between the AMI cases observed during 2013–2015 and the change in climate data using the 16 climate variables (for more information please see appendix 1).

The main purpose of clustering analysis is to classify objects into natural subgroups according to dissimilarity or similarity between the objects (units). However, in practice, it is not always know whether an object is a member of a cluster. Fuzzy clustering is a recommended method to deal with ambiguous classifications (31). This is because this method allows an object to belong to a cluster at a membership degree that makes it appropriate for real-world problems. In other words, it does not just assign an object to a cluster, it also calculates the membership degree of the object in that cluster.

The distance from fuzzy clusters to classical clusters was evaluated by Dunn’s partition coefficient. This coefficient indicates how ‘fuzzy’ the obtained cluster is. A value close to 1, indicates more appropriate to classify the data by the fuzzy clustering analysis. Moreover, the Silhouette coefficient *s(i)* and average silhouette coefficients *s̄(i)* were computed to measure the consistency within the clusters and assess the validity of clustering. Table 1 shows the evaluation criteria of the average silhouette coefficient developed by Kaufman and Rousseeuw (30) and Ozdamar (32).

**Table 1:** Criteria to Evaluate Average Silhouette Coefficient

***Average Silhouette Coefficient*** *s̄(i)*	***Interpretation***
−1≤*s̄*(*i*)≤0.25	Appropriate aggregation structure is weak
0.26≤*s̄*(*i*)≤0.50	Missing/Artificial clustering structure
0.51≤*s̄*(*i*)≤0.70	proper / reasonable clustering structure
0.71≤*s̄*(*i*)≤1.00	There is a strong clustering structure

## Results

The distribution of AMI numbers to 12 months from 2013 to 2015 is reported in Table 2. The results of conducting a Chi-Square test showed that the distribution of AMI cases over months was not homogeneous so that AMI cases in March, April, and May were more frequent than other months.

**Table 2:** The number of patients per month

***Months***	***1***	***2***	***3***	***4***	***5***	***6***	***7***	***8***	***9***	***10***	***11***	***12***
Patient Numbers	512	464	741	828	616	454	462	490	443	427	471	469

The results of performing the fuzzy clustering analysis are reported in Table 3. The *s̄*(*i*) and *s̅*(*i*) values were in acceptable ranges. The membership coefficients of the months indicated two groups: one was [January, February, June, July, August, September, October, November, and December] and the other one was [March, April, and May].

**Table 3:** Membership coefficients and average and overall average silhouette coefficient for each months

***Months***	***Membership Cluster 1***	***Coefficient Cluster 2***	***Cluster***	***Neighbour Cluster***	***Silhouette coefficient*** *s(i)*	***Average Silhouette coefficient*** *s̄(i)*	***Average Silhouette coefficient of total data*** *s̅(i)*
7 (Jul)	0.928	0.071	1	2	0.803	0.746	0.662
9 (Sep)	0.919	0.080	1	2	0.801		
12 (Dec)	0.912	0.087	1	2	0.782		
11 (Nov)	0.908	0.091	1	2	0.781		
10 (Oct)	0.882	0.117	1	2	0.765		
8 (Agu)	0.886	0.113	1	2	0.749		
6 (Jun)	0.860	0.139	1	2	0.725		
2 (Feb)	0.820	0.179	1	2	0.670		
1 (Jan)	0.810	0.189	1	2	0.637		
3 (Mar)	0.075	0.926	2	1	0.621	0.411	
4 (Apr)	0.111	0.888	2	1	0.599		
5 (May)	0.382	0.617	2	1	0.014		

**Fig. 1:**
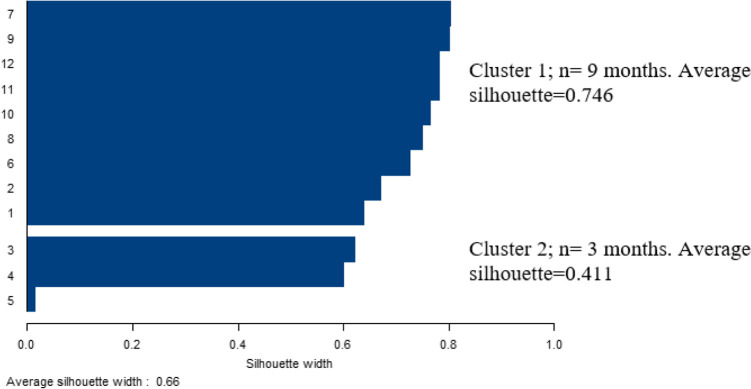
Average silhouette figure for best classification

Based on Fig. 1, the value showed that while both clusters had an appropriate clustering structure, the cluster including nine months was more stable and homogenous than the cluster including Mar, Apr, and May. Dunn’s partition coefficient and Normalized Dunn’s partition coefficient were 0.778 and 0.555 respectively supporting the goodness-of-fit of the analysis. The average silhouette width value of the total data (0.663) indicated that the classification of the fuzzy clustering analysis was quite accurate.

**Fig. 2:**
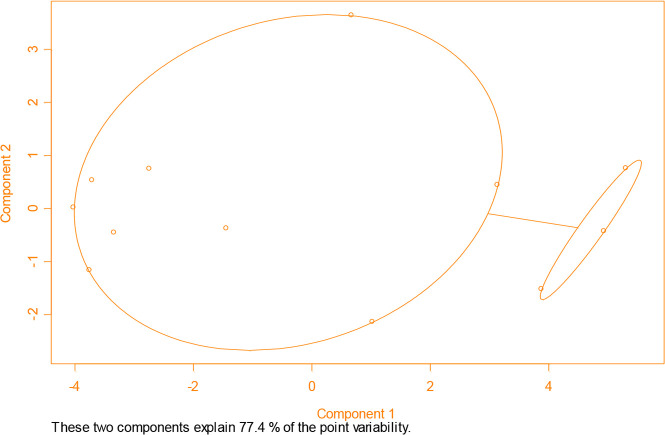
Results of fuzzy classification

Figure 2 displays the obtained two-clustered structure. Mar, Apr, and May were clustered in one group and the rest in the other group. Moreover, the results showed that 77.4% of the variation can be explained by the model.

## Discussion

According to the obtained results from the fuzzy clustering analysis performed in this study, when 12 months of the year were classified using 16 climatic variables, Jan, Feb, June Jul, Aug, Sep, Oct, Nov, and Dec are clustered together (Cluster 1) and Mar, Apr, and May are clustered together (Cluster 2). Besides, when the distribution of AMI numbers by months is examined, the AMI cases occur at most in March, April, and May (*P* < 0.05). Accordingly, changes in the climate data trigger AMI. In other words, AMI is affected by changes in the climate.

In recent decades, studies have been done on various mechanisms and predisposing factors for AMI (33–35). Some of these studies assessed the effects of climate conditions on AMI, and have stated that meteorological parameters and seasonal changes can play an important role in its incidence (36, 37). Insofar as approximately 4% of the incidence of AMI is related to several kinds of meteorological parameters (8), such as air temperature, humidity, and wind speed air pressure (21, 22, 38). In this regard, the role of seasonal changes with the incidence of AMI, numerous findings have also been reported. Most of them are consistent with our finding that AMI often occurs in the winter and spring (26, 39–42). Contrary to our findings, the study indicated an increase of AMI incidence in the summer when we face the height of temperature and humidity and the lower pressure of the atmosphere (43). Physiological stressors such as the activation of the sympathetic system, the increasing risk of infection in cold climates (i.e. influenza and air pollution) (26, 44, 45) and enhancing the immune response, high blood pressure and inactivity and immobility (46, 47) are related to incidence of AMI in the winter.

The three months of spring season in Caspian coastal areas are accompanied by sever changes in atmospheric parameters such as air temperature, humidity, and wind. This classification can be used to describe the variation of weather patterns that are useful for anticipating the incidence of AMI. These results provide information that can be used to manage, control, and provide the essential diagnostic, hospital, and remedial services to AMI patients during the months with highest probability of AMI incidences. Moreover, identifying the most prevalent time of the disease can be of a great help to effectively organize and distribute professional teams and care facilities during different seasons.

### Limitations and Recommendations

Same as most studies, this study had several limitations. 1) Using existing medical records that were collected for diagnosis and treatment and not specifically for the purpose to answer the research aims. Therefore, overreporting, underreporting, and errors in reporting may result in misclassification in the current study; 2) Lack of access to the details of all patient records (e.g. type of AMI, BMI, blood pressure, past medical history, BUN, Creatinine) which could have helped this study evaluate the results in the context of such other risk factors; 3) Absence of patients who died before reaching the hospital or patients that were not admitted (outpatients). Therefore, more detailed results about the incidence of AMI regarding seasonal changes can help in decreasing the prevalence. Future studies with samples from different populations and also longitudinal designs are suggested to verify the findings of this study. Future studies are recommended that incorporate more details about patients and include wider climate areas (such as warm and dry as well as cold and dry) into their analysis.

By identifying regions where adverse weather conditions threaten the life safety of people at higher risk for AMI, communities can be alerted and prepared to allocate needed resources and equipment to respond to higher AMI incidence and demand for services. Those at higher risk for AMI can be educated and counseled to take specific steps, whenever possible, to remain indoors and to avoid overexertion during times of danger.

## Conclusion

Climate changes were found to trigger AMI. Especially from cold weather to hot weather in March, April and May, was found to be significant increase in AMI. Therefore, AMI patients and emergency treatment service personnel should be more vigilant and prepared in Mar, Apr, and May.

## Ethical considerations

Ethical issues (Including plagiarism, informed consent, misconduct, data fabrication and/or falsification, double publication and/or submission, redundancy, etc.) have been completely observed by the authors.
